# Morphological Alterations and Increased S100B Expression in Epidermal Langerhans Cells Detected in Skin from Patients with Progressive Vitiligo

**DOI:** 10.3390/life11060579

**Published:** 2021-06-18

**Authors:** Fei Yang, Lingli Yang, Lanting Teng, Huimin Zhang, Ichiro Katayama

**Affiliations:** 1 Department of Pigmentation Research and Therapeutics, Graduate School of Medicine, Osaka City University, Osaka 5450051, Japan; youhi0613@gmail.com (F.Y.); tenrantei@gmail.com (L.T.); espaikoffice@gmail.com (I.K.); 2 Department of Dermatology, Course of Integrated Medicine, Graduate School of Medicine, Osaka University, Osaka 5650871, Japan; 3 Department of Dermatology, Shuguang Hospital Affiliated to Shanghai University of Traditional Chinese Medicine, Shanghai 200025, China

**Keywords:** Langerhans cells, vitiligo, leukoderma, Birbeck granule, S100B

## Abstract

The role of Langerhans cells (LCs) in vitiligo pathogenesis remains unclear, with published studies reporting contradictory results regarding the quantity of LCs and no data on the features of LCs in vitiligo. Here, we aimed to analyze the presence, density, and morphological features of LCs in the epidermis of patients with vitiligo. Skin biopsies were stained for LCs using anti-CD1a/anti-langerin antibodies and analyzed by immunocytochemistry with light and electron microscopy. Compared with healthy controls, we detected significantly increased numbers of epidermal LCs in lesional skin from vitiligo in the progressive state. These LCs exhibited striking morphological alterations, including an elevated number of dendrites, with increased length and more branches than dendrites from controls. Ultrastructure examination via immuno-electron microscopy revealed markedly reduced Birbeck granules (BGs) and shorter BG rods in LCs from progressive vitiligo, with higher expression of langerin. Additionally, expression of S100B, the activity biomarker of vitiligo, was increased in these LCs. This work provides new insight on the cellular composition of LCs in vitiliginous skin, revealing altered morphology and increased LC numbers, with elevated S100B expression. Our data suggest LCs might play a critical role in vitiligo pathogenesis and thus may represent a novel therapeutic target for this disease.

## 1. Introduction

The skin is the largest organ in the human body, and it plays a critical role in protecting from environmental assaults. As such, the skin contains an intricate network of immune cells that function to defend against pathogen invasion and protect the body from foreign substances [1]. Critically, however, an imbalance in immune homeostasis may lead to a disease state.

Vitiligo is an acquired pigmentary disease, characterized by white spots on the skin that arise due to the absence of functional melanocytes in the epidermis. It is the most common disorder of pigmentation, affecting 1–2% of the world’s population [2,3]. Notably, however, despite its prevalence and the fact that vitiligo has been known for thousands of years, the cause is still unclear [3]. Many hypotheses have been proposed, including autoimmune, genetic, melanocytorrhagy, and neural etiologies, but no single model has been able to explain the full disease spectrum, and the pathogenesis of vitiligo remains puzzling [4].

Recently, it has been suggested that disruption of immune homeostasis is involved in vitiligo pathogenesis, although more studies are needed to test this hypothesis [5]. Clinically, doctors have noted that depigmented skin from patients with vitiligo does not develop contact dermatitis in response to sensitization with dinitrofluorobenzene (DNFB) [6,7], although pigmented skin from vitiligo patients responded normally. These findings suggest that the immune response is disrupted in depigmented vitiligo skin.

Langerhans cells (LCs) are skin-resident antigen-presenting cells that are characterized by CD1a and CD207/langerin expression, as dendritically shaped cells located in the epidermis. LCs play an important role in local defense and the establishment of immune responses [8,9]. Within the epidermis, LCs are positioned in the suprabasal layer and communicate with keratinocytes and melanocytes [10]. Epidermal LCs have been proposed to contribute to vitiligo, although their exact role remains unclear. In particular, a number of studies have reported contradictory results regarding the quantity of epidermal LCs in vitiligo, and there are no published data describing their features in vitiliginous skin [11,12,13].

S100 calcium-binding protein B (S100B), a member of S100 protein family, is synthesized and secreted by astrocytes, melanocytes, and LCs [14,15]. S100B levels are positively associated with age, as well as neurodegenerative [16], neuroinflammatory [15], and various skin inflammatory diseases [17]. Recently, S100B has also been suggested to act as a potent biomarker for vitiligo activity [18,19,20,21].

In this study, to investigate the role of LCs in vitiligo pathogenesis, we assessed the number and morphology of LCs in the epidermis of vitiliginous skin. Compared with healthy controls, we found that lesional skin from patients with vitiligo in the progressive state contains a marked increase in the quantity of epidermal LCs, and these show an altered morphology, with significant increases in the number of dendrites and dendrite length, fewer Birbeck granules (BGs), and shorter BGs rods expressing elevated levels of langerin. Furthermore, LCs from these patients show increased expression of S100B. Thus, our work provides new insight on the cellular composition of LCs in vitiliginous skin and suggests that these cells may play a role in disease pathogenesis.

## 2. Materials and Methods

### 2.1. Human Skin Specimens

Lesional skin biopsy specimens from patients with confirmed non-segmental vitiligo and in the progressive state (*n* = 8), patients with non-segmental vitiligo in the stable state (*n* = 10), patients with rhododendrol-induced leukoderma (RDIL) in the progressive state (*n* = 13) (Table 1), and samples from corresponding sites of healthy control subjects (*n* = 5: 2 from forehead, 1 from back of hand, 1 from abdomen, and 1 from neck) were used in this study. Progressive state was defined as the development of new lesions or extension of preexisting lesions in 6 months, and stable state was defined as no increase in size of existing lesions and absence of new lesions in 6 months. Patients with past treatments (systemic PUVA, excimer light, tarcolimus, systemic or topical steroid, etc.) were not included in this study. Written informed consent was obtained from all participants prior to study inclusion. The study was approved by the ethics committee of the Osaka City University Faculty of Medicine (No. 4152) and Osaka University Faculty of Medicine in Japan (No. 10339).

### 2.2. Human Fluorescent Immunohistochemistry Staining

Skin tissue samples were fixed in 10% formaldehyde and embedded in paraffin. These were sliced into 5 μm sections, deparaffinized, dehydrated, and heat-incubated with antigen retrieval solution (Target Retrieval Solution, PH9; Dako, Agilent, Santa Clara, CA, USA) for approximately 15 min. After blocking with horse serum (Vector Laboratories, Marion, Burlingame, CA, USA), sections were incubated with primary antibodies specific for CD1a (1:100 dilution, #M357101, Dako, Santa Clara, CA, USA), Melan-A (1:100 Dilution, #ab731, Abcam, Cambridge, UK), and S100B (1:100 Dilution, #HPA015768, Sigma-Aldrich, St. Louis, MO, USA) overnight at 4 °C and then incubated with a secondary antibody for 1 h (1:500 dilution; anti-mouse IgG1 AlexFluor 488 for CD1a, anti-mouse IgG2b AlexFluor 555 or anti-mouse IgG2b AlexFluor 647 (pseudocolor white) for Melan-A, anti-rabbit IgG AlexFluor 555 for S100B; Invitrogen, Thermo Fisher Scientific, Waltham, MA, USA) [22]. Sections were counterstained with Hoechst 33342 at a 1:500 dilution (Invitrogen, Carlsbad, CA, USA), and stained sections were visualized using a Biozero 8100 confocal microscope (Keyence Corporation, Osaka, Japan).

### 2.3. Human Pre-Embedding Immuno-Electron Microscopy

Pre-embedding immuno-electron microscopy was performed as detailed previously [23], with slight modification. Briefly, finely minced pieces of skin tissue were fixed in 4% paraformaldehyde for 2 h at room temperature, followed by embedding in optimal cutting temperature (OCT) compound. Frozen samples were cut into 10 μm sections and permeabilized in phosphate buffer (PB), containing 0.25% saponin for 30 min. Sections were blocked for 30 min in PB, containing 0.1% saponin, 10% bovine serum albumin, 10% normal goat serum, and 0.1% cold water fish skin gelatin, and exposed overnight to rabbit anti-langerin primary antibodies (#13650, Cell Signaling Technology, Danvers, MA, USA) in blocking solution. Specimens were then incubated with colloidal gold-conjugated goat anti-rabbit IgG (1.4 nm diameter, #2003, Nanoprobes, Yaphank, NY, USA) in blocking solution for 2 h, and the signal was intensified with the GoldEnhance EM kit (#2113, Nanoprobes, Yaphank, NY, USA) for 4 min at room temperature. The specimens were post-fixed in 1% OsO_4_, containing 1.5% potassium ferrocyanide, and then dehydrated in a series of graded ethanol solutions and embedded in epoxy resin. Ultrathin sections were collected and stained with uranyl acetate and lead citrate, and images were acquired with a Hitachi H7650 electron microscope, equipped with an Advanced Microscopy Techniques charge-coupled device-based camera system.

### 2.4. Quantitative and Morphometric Analyses

All immunohistochemistry-stained sections were photographed in 5 non-contiguous random grids under high-power magnification fields (400×). The quantitative and morphometric analyses were performed using Image J software (version 1.53j, National Institutes of Health, Bethesda, MD, USA). CD1a-positive cells and all the Hoechst 33342-positive cells in epidermis were automatically counted with Image J software by establishing a color density threshold [24]. Then, the average ratio of LCs to the total epidermal cells were calculated. The number of dendrites or BGs in LCs were counted using the “Multi-point Tool”, and the length of dendrites or BGs were measured using the “Straight Tool” in ImageJ.

In all immunoelectron microscopy sections, 10 LCs in 10 non-contiguous fields were photographed. Then, the number of BGs in LCs were counted using the “Multi-point Tool”, the length of BGs was measured using the “Straight Tool”, and the number of Langerin colloids in LCs were quantified automatically in ImageJ.

### 2.5. Statistical Analysis

Data are presented as the mean ± standard deviation (SD). Comparisons were performed using Kruskal–Wallis and Mann–Whitney U tests, and correction for multiple comparisons used Bonferroni (Microsoft Excel, version 16.0, Microsoft Corp., Redmond, WA, USA); *p*-values < 0.05 were considered statistically significant.

## 3. Results

### 3.1. Epidermal LCs Are Increased in Lesional Skin from Progressive Vitiligo Relative to Normal Skin

**Figure 1:**
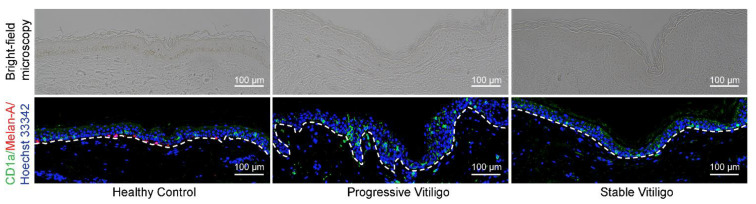
Epidermal Langerhans cells (LCs) are increased in lesional skin from progressive vitiligo relative to stable vitiligo and normal skin. Skin sections from healthy controls (*n* = 5), non-segmental vitiligo in the progressive state (Progressive Vitiligo, *n* = 8), and non-segmental vitiligo in the stable state (Stable Vitiligo, *n* = 10) were stained with anti-CD1a (green) and anti-Melan-A (red) antibodies. Nuclei were stained in blue with Hoechst 33342. Representative images are shown, and the corresponding bright-field microscopy images are displayed in the upper panels. Scale bar: 100 μm.

Previous studies investigating the quantity of LCs in vitiligo-affected skin have reported contradictory results, with some observing a reduction in the percentage of epidermal LCs [13] and others reporting an increase in epidermal LCs [11,12]. To resolve this issue and determine whether there are changes in LC numbers in the epidermis of patients with vitiligo, we obtained formalin-fixed paraffin-embedded tissue from eight lesional skin samples of non-segmental vitiligo in the progressive state, ten lesional skin samples of non-segmental vitiligo in the stable state, and five normal skin samples. We then visualized LCs by fluorescence immunohistochemistry staining with anti-CD1a antibodies (shown in green); melanocytes were visualized by staining with anti-Melan-A antibodies (shown in red) (Figure 1). As expected, we observed a disappearance of melanocytes and epidermal pigment granules in tissue from both progressive and stable vitiligo (Figure 1). In contrast, we detected increased LC staining in the epidermis of progressive vitiligo samples, as compared with both normal skin and stable vitiligo (Figure 1). These results indicate an increase in epidermal LCs in lesional skin from progressive vitiligo, suggesting that LCs may be involved in disease progression.

### 3.2. Increased Number of Dendrites with Increased Length Detected in LCs of Lesional Skin from Progressive Vitiligo

Because an increase in epidermal LCs was observed in progressive vitiligo rather than stable vitiligo, we mainly focused the remainder of our investigation on LCs in progressive vitiligo. In addition, it was recently shown that RDIL, a novel pigmentary disorder that is associated with the disappearance of melanocytes and appearance of white patches on the skin, has phenotypic as well as pathophysiological similarity to vitiligo [25,26]. Therefore, we also investigated LCs in 13 biopsies from progressive RDIL.

**Figure 2:**
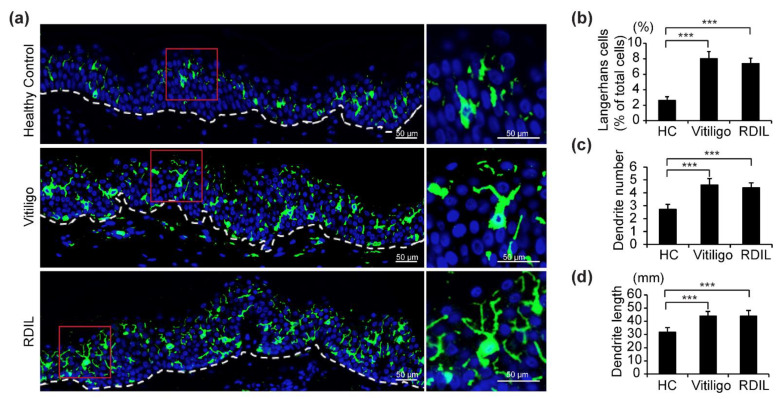
Increased number of LCs, dendrites per LC, and dendrite length in lesional skin from progressive vitiligo and progressive rhododendrol-induced leukoderma (RDIL). (**a**) Skin sections from healthy controls (*n* = 5), non-segmental vitiligo in the progressive state (Progressive Vitiligo, *n* = 8), and RDIL in the progressive state (RDIL, *n* = 13) were stained with anti-CD1a antibodies (green). Nuclei were stained in blue with Hoechst 33342. Representative images are shown, with higher-magnification images corresponding to the area surrounded by the red rectangles in left panels shown at right. Scale bar: 50 μm. Quantification of LCs (percentage of total cells in epidermis) (**b**), number of dendrites per LC (**c**), and length of dendrites (**d**) in LCs. Data in (**b**–**d**) are shown as the means ± standard deviation (SD). *** *p* < 0.01.

Fluorescence immunohistochemistry staining with anti-CD1a antibodies (shown in green) of samples from progressive vitiligo, progressive RDIL, and normal skin revealed that LCs in the epidermis of both progressive vitiligo and RDIL are greatly increased relative to normal skin, and that these cells are large and polygonal in shape, with more dendrites/branches and longer dendrites, compared with healthy controls (Figure 2a). We then quantified the number of total LCs, dendrites per LC, and length of dendrites in these stained sections. We found that in healthy controls, epidermal LCs constitute 3% of the cell population in the epidermis. However, a significant increase of approximately 2.5–3 times that amount is observed in the epidermis of progressive vitiligo (about 8%) and RDIL (about 7%) (Figure 2b). In addition, the number of dendrites per LC (Figure 2c) and dendrite length (Figure 2d) are significantly increased in progressive vitiligo and progressive RDIL relative to normal tissue.

### 3.3. Decreased BGs and Shorter BG Rods with Elevated Langerin Expression Detected in LCs of Progressive Vitiligo

We next investigated the ultrastructural features of the LCs in samples from progressive vitiligo using immuno-transmission electron microscopy (TEM). In total, three samples from progressive vitiligo and three samples from healthy controls were stained with anti-langerin (CD207) antibodies and subjected to immuno-electron microscopy analyses. We chose to stain for langerin as this is another specific marker for LCs and a crucial component of BGs, a unique rod-shaped organelle found in LCs [27].

**Figure 3:**
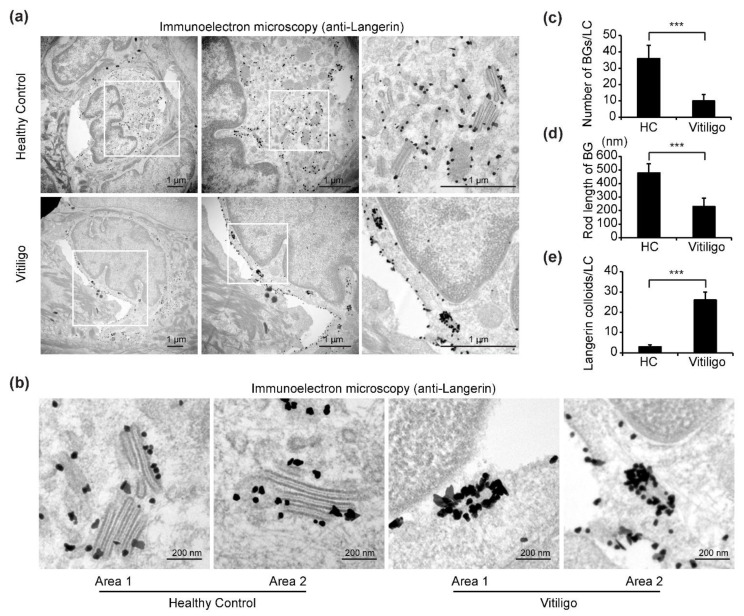
Immuno-transmission electron microscopy (TEM) reveals altered Birbeck granule (BG) morphology in epidermal LCs from progressive vitiligo. Skin sections from healthy controls (*n* = 3) and non-segmental vitiligo in a progressive state (Progressive Vitiligo, *n* = 3) were stained with anti-langerin antibodies, and immuno-TEM was performed. (**a**) Representative TEM images are shown; areas framed in white rectangles are enlarged in right panels. Small black dots represent 30–50 nm sized colloidal gold (enhanced from 1.4 nm nanogold) particles labeling langerin. Scale bar: 1 μm. (**b**) Representative high-power TEM images of BGs in healthy controls and progressive vitiligo. Scale bar: 200 nm. Quantification of BGs per LC (**c**), rod length of BGs (**d**), and langerin colloids per LC (**e**) in progressive vitiligo and healthy controls. Data in (**c**–**e**) are shown as means ± SD. *** *p* < 0.01.

In immuno-TEM images, cells with hallmark features of LCs, including a cleaved or folded nucleus, clear cytoplasm devoid of tonofilaments or desmosomes, absence of melanosomes or pre-melanosomes, and with small (30–50 nm) dark dots indicating gold-labeled langerin staining were selected for our analyses (Figure 3a). In samples from healthy controls, numerous BGs with typical features, including an elongated rod or tennis racket-resembling shape, a laminar structure, and surface accumulation of langerin, were observed in the cytoplasm of LCs (Figure 3a). Remarkably, however, in samples from progressive vitiligo, it was difficult to find typical BGs; in some areas of the cytoplasm, langerin accumulation could be detected, but BG morphology was clearly abnormal. In particular, the rods were notably shorter and surface accumulation of langerin was massively increased compared with what is observed in typical BGs from control samples (Figure 3a,b). Further, quantitative analyses reveal a significant reduction in both total BGs per LC (Figure 3c) and BG rod length (Figure 3d), as well as a significant increase in langerin expression (Figure 3e) in epidermal LCs from progressive vitiligo.

### 3.4. Increased Expression Level of S100B in Epidermal LCs from Progressive Vitiligo

**Figure 4:**
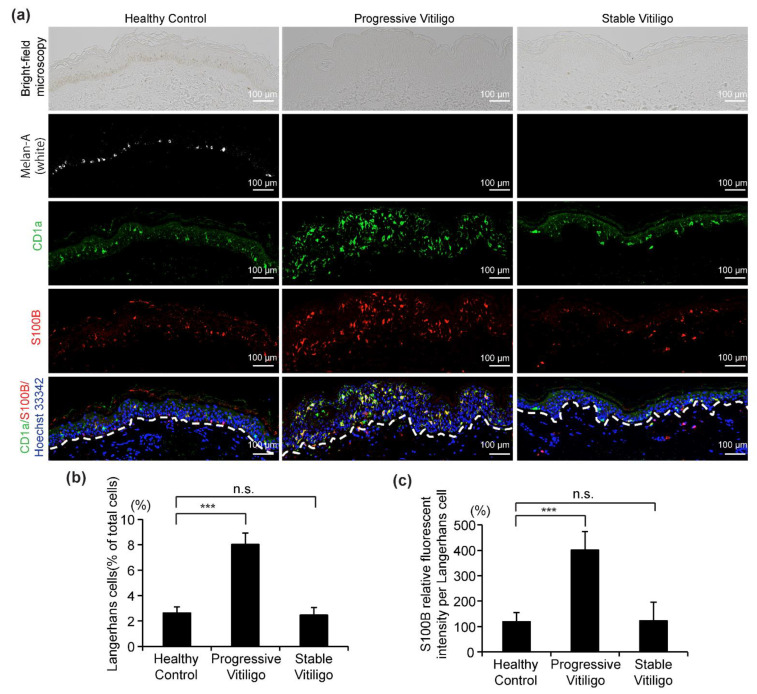
Increased S100B expression level in LCs from the skin of subjects with progressive vitiligo. (**a**) Skin sections from healthy control (*n* = 5), non-segmental vitiligo in a progressive state (Progressive Vitiligo, *n* = 8), and non-segmental vitiligo in a stable state (Stable Vitiligo, *n* = 10) were stained with anti-Melan-A (white), anti-CD1a (green), and anti-S100B (red) antibodies. Nuclei were stained in blue with Hoechst 33342. Representative images are shown, and the corresponding bright-field microscopy images are displayed in the upper panels. Scale bar: 100 μm. (**b**) Quantification of LCs (percentage of total cells in epidermis); (**c**) quantification of S100B expression level in LCs (relative fluorescent intensity compared with healthy control). Data in (**b**,**c**) are shown as means ± SD. *** *p* < 0.01, n.s., no statistical significance.

Recently, S100B was suggested as a potential disease activity maker in non-segmental vitiligo [18,28]. In these studies, it was proposed that S100B is released from damaged melanocytes [18,28]. However, the S100B protein is normally present in the epidermis in LCs, and in fact, it is also routinely used as a marker for LCs. Here, to determine whether S100B expression is elevated in LCs from vitiligo, we analyzed sections from eight lesional skin samples of non-segmental vitiligo in a progressive state, ten non-segmental vitiligo in a stable state, and five healthy controls using four-color fluorescent immunohistochemistry to stain for melanocytes (white), LCs (green), S100B (red), and nuclei (blue) (Figure 4a). Consistent with our results in Figure 1, we observed the disappearance of melanocytes and epidermal pigment granules both in progressive vitiligo and stable vitiligo, with more LCs detected in the epidermis of progressive vitiligo, compared with normal skin and stable vitiligo (Figure 4b). In addition, we detected markedly increased S100B expression in the epidermis of skin from progressive vitiligo, and this was mainly observed in LCs. We further note that S100B expression level was much higher in LCs of progressive vitiligo than in LCs from either stable vitiligo or healthy controls (Figure 4c). These results suggest that the LCs in progressive vitiligo might be distinct not only in their density and morphology, but also in regard to their cellular function.

## 4. Discussion

A number of previous studies have investigated LCs in vitiligo and reported contradictory findings. Itoi et al. [11] observed an increase in, as well as activation of, LCs from progressive non-segmental vitiligo. Studies by Prignano et al. and Seite et al. [12,29] reported that narrow-band ultraviolet B (NB-UVB) and UVA1 treatment reduced LC density, redistributed main LCs, and slightly altered morphology of LCs in vitiligo. However, recently, in a study with 20 vitiligo patients and 10 age- and sex-matched controls, Shoeib et al. [13] reported a reduction in LC percentage in skin affected by vitiligo and found no statistical change in LC percentage after NB-UVB therapy. Thus, the quantity and role of epidermal LCs in vitiligo has remained unclear.

In the current study, we aimed to address this question by analyzing LCs in skin sections from progressive and stable vitiligo, using confocal and immuno-electron microscopy to assess cell numbers and morphology, as well as expression of the vitiligo activity biomarker, S100B. We found that, in comparison with healthy controls and stable vitiligo, LCs in the epidermis of progressive vitiligo are significantly increased and show morphological alterations, as evidenced by both by confocal fluorescent microscopy and immuno-electron microscopy. In addition, these LCs display higher expression of S100B.

Vitiligo is an acquired, chronic skin pigmentary disorder, characterized by progressive loss of melanin and melanocytes in the epidermis [3]. The precise cause remains unknown, but an increasing number of observations highlight the important role of cellular immunity in the pathogenesis of this disease [5,30].

LCs were first identified in 1868 by Paul Langerhans [31]. These cells are derived from bone marrow and comprise approximately 2–3% of cells within the epidermis of normal human skin [29]. Epidermal LCs are known to play critical roles in contact hypersensitivity reactions and other defense mechanisms against skin tumors [32]. In the present study, we found that LCs make up about 3% of cells within the epidermis of healthy skin, whereas in progressive vitiligo, we observe a significant increase in these cells in epidermal tissue, with LCs comprising about 8% of the total cell population. These data suggest that LC-mediated cellular immunity may be critical for the progression of vitiligo.

LCs are characterized by their typical dendritic morphology and the characteristic “tennis racket-shaped” BGs in their cytoplasm [32]. A number of previous studies have reported decreased numbers of LCs that exhibit an altered morphology in some squamous cell carcinomas and basal cells carcinomas (BCCs) of the skin; in particular, these LCs were mainly round or oval, rather than dendritic [33,34,35,36]. The authors postulated that alterations in LC numbers and morphology in BCC were likely secondary to changes in the local environment and suggested that they might contribute to the development of skin tumors [33,34,35,36]. In the present study, we noted remarkable morphological changes in epidermal LCs from progressive vitiligo. Specifically, these cells have more and longer dendrites, with fewer BGs in their cytoplasm and shorter BG rods.

Normal LCs have been classified into two types; Type 1 LCs are pyramidal in shape and contain numerous BGs and long-branched dendritic processes and Type 2 LCs are spherical in shape with fewer BGs and shorter dendritic processes [27]. Here, we found that LCs in progressive vitiligo are more dendritic, with more branches and longer dendrites. Thus, to a certain extent, they have features that are similar to Type 1 LCs. However, LCs from progressive vitiligo have fewer BGs and shorter rods in their BGs. In previous reports, it was shown that cytokines, growth factors, and keratinocyte-derived hormones can influence LC phenotype and function [37]. Our data suggest that the altered BGs in LCs from progressive vitiligo might represent a pathognomonic feature, resulting from the local environment, and that these LCs might, in turn, contribute to the abnormal immune response observed in progressive vitiligo.

In addition to the cell surface marker CD1a, langerin is also used as a marker for identifying LCs via immunohistochemical staining [32]. In the present study, we performed immuno-electron microscopy with gold-labeled langerin and observed increased accumulation of langerin in BGs within the LCs from progressive vitiligo relative to those from healthy controls. Langerin has been proposed to induce BG formation, and expression of this protein is regulated by transforming growth factor (TGF)-β1, tumor necrosis factor (TNF)α, and the tissue environment [38,39]. We speculate that in LCs from progressive vitiligo, increased accumulation of langerin in BGs might result from a disruption in BG formation or feedback from either the altered BGs or the aberrant tissue environment.

S100B is expressed in dendritic cells and cells with a neurogenic origin, and increased levels of this protein have been reported in neurological diseases, psoriasis, and other inflammatory disorders [14,15,17,40]. Recently, S100B was also suggested as a potent biomarker of activity in progressive vitiligo [18,19,20,21]. Furthermore, in a monobenzone-induced vitiligo mouse model, S100B inhibition was found to alleviate depigmentation in mouse skin [28]. In these previous reports, melanocytes were thought to be the source of S100B. However, in the present study, we detected markedly higher S100B expression in LCs from progressive vitiligo than in LCs from healthy controls or stable vitiligo. The S100B protein is known to play diverse roles in many cellular processes, including regulation of protein phosphorylation, cell proliferation and motility, cell cycle progression, transcription, cell differentiation, and calcium homeostasis [41]. Our data suggest the possibility that S100B in LCs might also be involved in the activity or progression of vitiligo.

The histopathological and morphological data obtained in this study may help to answer a number of unresolved questions on the pathogenesis of vitiligo and may suggest a role for LCs in the progression of this disease. Critically, however, the precise functions of LCs in progressive vitiligo have yet to be determined, and further studies will be required to elucidate the mechanisms underlying our observed morphological changes.

## 5. Conclusions

In conclusion, we find that epidermal LCs in lesional skin from progressive vitiligo are largely increased in number and display morphological alterations relative to LCs from stable vitiligo or healthy skin. Furthermore, these LCs show altered BG morphology and increased expression of S100B. Our data suggest that LCs might play a role in the pathogenesis of vitiligo and therefore may represent a possible therapeutic target for the development of new treatments.

## Figures and Tables

**Table 1:** Clinical characteristics of patients.

Patient No.	Sex	Age (y)	Duration (y)	Type	Lesion Site	Activity
1	F	49	2	Non-segmental vitiligo	Back of hand	Stable
2	M	79	7	Non-segmental vitiligo	Forehead	Progressive
3	F	69	1	RDIL *	Forehead	Progressive
4	F	55	10	Non-segmental vitiligo	Abdomen	Progressive
5	F	66	0.4	RDIL	Back of hand	Progressive
6	F	63	4	Non-segmental vitiligo	Lumbar	Stable
7	F	74	0.6	RDIL	Forehead	Progressive
8	F	63	1	RDIL	Abdomen	Progressive
9	F	38	0.5	RDIL	Neck	Progressive
10	F	59	0.8	RDIL	Back of hand	Progressive
11	M	80	0.5	Non-segmental vitiligo	Forehead	Progressive
12	F	70	6	Non-segmental vitiligo	Chin	Stable
13	F	43	3	Non-segmental vitiligo	Neck	Stable
14	F	66	8	Non-segmental vitiligo	Chest	Stable
15	F	59	3	Non-segmental vitiligo	Arm	Stable
16	F	60	4	Non-segmental vitiligo	Leg	Progressive
17	F	64	5	Non-segmental vitiligo	Shoulder	Stable
18	M	15	1	Non-segmental vitiligo	Face	Progressive
19	F	84	1	RDIL	Neck	Progressive
20	M	35	5	Non-segmental vitiligo	Chest	Progressive
21	F	42	0.3	RDIL	Face	Progressive
22	F	48	0.5	RDIL	Neck	Progressive
23	M	48	4	Non-segmental vitiligo	Face	Stable
24	M	58	3	Non-segmental vitiligo	Wrist	Stable
25	M	36	5	Non-segmental vitiligo	Abdomen	Progressive
26	F	35	0.5	RDIL	Back of hand	Progressive
27	F	71	1	RDIL	Neck	Progressive
28	F	67	0.3	RDIL	Forehead	Progressive
29	F	38	3	Non-segmental vitiligo	Forehead	Progressive
30	F	66	1	RDIL	Abdomen	Progressive
31	F	77	3	Non-segmental vitiligo	Back of hand	Stable

* RDIL: rhododendrol-induced leukoderma.

## Data Availability

The data presented in this study are available on request from the corresponding author.
